# XIAP over-expression is an independent poor prognostic marker in Middle Eastern breast cancer and can be targeted to induce efficient apoptosis

**DOI:** 10.1186/s12885-017-3627-4

**Published:** 2017-09-11

**Authors:** Azhar R. Hussain, Abdul Khalid Siraj, Maqbool Ahmed, Rong Bu, Poyil Pratheeshkumar, Alanood M. Alrashed, Zeeshan Qadri, Dahish Ajarim, Fouad Al-Dayel, Shaham Beg, Khawla S. Al-Kuraya

**Affiliations:** 1 Human Cancer Genomic Research, King Faisal Specialist Hospital and Research Cancer, MBC#98-16, P.O. Box 3354, Riyadh, 11211 Saudi Arabia; 2 AlFaisal University, Riyadh, Saudi Arabia; 3 Oncology Center, King Faisal Specialist Hospital and Research Center, Riyadh, Saudi Arabia; 4 Department of Pathology, King Faisal Specialist Hospital and Research Center, Riyadh, Saudi Arabia

**Keywords:** Breast cancer, XIAP, Embelin, P-AKT, Apoptosis

## Abstract

**Background:**

Breast cancer is the most common cancer in females and is ranked second in cancer-related deaths all over the world in women. Despite improvement in diagnosis, the survival rate of this disease has still not improved. X-linked Inhibitor of Apoptosis (XIAP) has been shown to be over-expressed in various cancers leading to poor overall survival. However, the role of XIAP in breast cancer from Middle Eastern region has not been fully explored.

**Methods:**

We examined the expression of XIAP in more than 1000 Middle Eastern breast cancer cases by immunohistochemistry. Apoptosis was measured by flow cytometry. Protein expression was determined by western blotting. Finally, in vivo studies were performed on nude mice following xenografting and treatment with inhibitors.

**Results:**

XIAP was found to be over-expressed in 29.5% of cases and directly associated with clinical parameters such as tumor size, extra nodal extension, triple negative breast cancer and poorly differentiated breast cancer subtype. In addition, XIAP over-expression was also significantly associated with PI3-kinase pathway protein; p-AKT, proliferative marker; Ki-67 and anti-apoptotic marker; PARP. XIAP over-expression in our cohort of breast cancer was an independent poor prognostic marker in multivariate analysis. Next, we investigated inhibition of XIAP using a specific inhibitor; embelin and found that embelin treatment led to inhibition of cell viability and induction of apoptosis in breast cancer cells. Finally, breast cancer cells treated with combination of embelin and PI3-kinase inhibitor; LY294002 synergistically induced apoptosis and caused tumor growth regression in vivo.

**Conclusion:**

These data suggest that XIAP may be playing an important role in the pathogenesis of breast cancer and can be therapeutically targeted either alone or in combination with PI3-kinase inhibition to induce efficient apoptosis in breast cancer cells.

**Electronic supplementary material:**

The online version of this article (10.1186/s12885-017-3627-4) contains supplementary material, which is available to authorized users.

## Background

Breast cancer is the most common cancer in females and despite improvement in treatment modality, the overall survival rate of breast cancer remains low [1]. Even though, incidence of breast cancer increases with age [2], it has been seen that there is trend towards an increase in incidence of breast cancer in younger women in western countries as well as Middle Eastern region [3–5]. In Saudi Arabia, breast cancer is the most common cancer in females as well as remains the major cause of morbidity and mortality within the female population [6]. One reason behind this increase in morbidity and mortality in breast cancer could be the strong-association with many aggressive molecular markers that tend to cause increased proliferation of cancer cells and impart resistance to conventional chemotherapy [7, 8]. These aggressive markers include dysregulated proteins of the survival pathways [8] and proliferative markers [9] that tend to make the tumor resistant to conventional chemotherapy, grow rapidly and spread to surrounding tissues and distant organs. For these reasons, there is an urgent need for identifying molecular targets that are either over-expressed or constitutively activated in breast cancer that can be therapeutically targeted.

Inhibitor of Apoptosis Proteins (IAPs) family is slowly emerging as viable therapeutic targets for the treatment of cancer because of their ability to be selectively over-expressed in various cancers as compared to their normal counterparts [10, 11]. Of the many members of the family, X-linked Inhibitor of Apoptosis Protein (XIAP) has been found to be the most promising target because XIAP is found to be over-expressed in a variety of cancers [12–15]. In addition, XIAP over-expression also leads to poor prognosis in many cancers including breast and thyroid cancer [14, 16]. Structurally, XIAP contains three tandem 80 amino acid repeats known as baculovirus IAP repeats (BIR) and a zinc ring domain that contains the E3 ligase ubiquitin activity thereby making XIAP susceptible to ubiquitination [17, 18]. The main role of XIAP is to disrupt and inhibit apoptosis by acting at caspase-3 and -7 via the second BIR domain and caspase-9 via the third BIR domain [19–21]. Because of the anti-apoptotic effect as well as its over-expressing potential in cancer cells as compared to its normal counterparts, XIAP is emerging as a potential therapeutic target for the management of cancer. There are several XIAP inhibitors have been reported and some are in clinical trial [22–24]. Embelin is the only natural, cell-permeable, non-peptide small molecule XIAP inhibitor reported so far [25, 26]. It selectively inhibits the growth of cancer cells and induces apoptosis, with non-toxic or low-toxic to normal cells [27]. Embelin binds to the BIR3 domain of XIAP and block the interaction of XIAP with caspases to promote apoptosis [28].

Survival of cancer cells is necessary for their propagation, invasion and migration leading to their disruptive behavior and damage to the normal working environment of the human body. This is usually achieved by not only over-expression of anti-apoptotic proteins but also by causing dysregulation of various signaling transduction pathways [29]. One pathway that is found to be dysregulated in many cancers is the PI3-kinase/AKT pathway whereby constitutive activation of survival protein, AKT promotes survival via inhibiting the apoptotic pathway, increased glucose metabolism and promote proliferation [30–32]. The PI3-kinase/AKT pathway has therefore been the target of many new experimental therapeutic agents because of its pro-survival and anti-apoptotic role in many cancers. However, the success of managing these cancers with single agents has been limited [33, 34]. On the other hand, PI3-kinase inhibitors have been more successful in combination with either other inhibitors or chemotherapeutic agents via sensitizing cancers cells to undergo apoptosis [35, 36].

In this study, we have investigated expression of XIAP in a large cohort of more than 1000 clinical breast cancer samples in tissue microarray (TMA) format by immunohistochemistry and determined the association of XIAP over-expression with various clinical parameters and molecular markers. This is followed by in vitro and in vivo targeting of XIAP in breast cancer cells using specific XIAP inhibitor, embelin, either alone or in combination with PI3-kinase/AKT inhibitor, LY294002 to assess cell viability, apoptosis and xenograft tumor regression.

## Methods

### Patient selection and tissue microarray (TMA) construction

Samples from 1009 breast cancer (BC) patients diagnosed between 1990 and 2011 were identified and selected from the tissue bio-repository of King Faisal Specialist Hospital and Research Centre (KFSHRC). Detailed clinico-pathological data, including survival data, were noted from case records. Follow-up was calculated from the date of resection of the primary tumor, and all surviving cases were censored for survival analysis on 31 December 2011. Three pathologists reviewed all tumors for grade and histological subtype. All BC samples were analyzed in a tissue microarray (TMA) format. TMA construction was performed from formalin-fixed, paraffin-embedded BC specimens and slides were processed and stained manually as described previously [37]. Briefly, tissue cylinders with a diameter of 0.6 mm were punched from representative tumor areas of a ‘donor’ tissue block using a semi-automatic robotic precision instrument and brought into 6 different recipient paraffin blocks each containing between 133 and 374 individual samples. A block containing normal and tumor tissue from multiple organ sites was used as control. Institutional Review Board (IRB) and Research Ethics Committee (REC) of KFSHRC approved the study under the Project RAC#2040 004 on BC archival clinical samples along with a waiver of consent and Project RAC#2110 025 for animal studies.

### Immunohistochemistry

Primary antibody clones and their dilutions used for IHC are given in Additional file 1: Table S1. XIAP, PARP, Ki-67 and p-AKT expression were analyzed by immunohistochemistry on a TMA as described before [12]. X-tile plots were constructed for assessment of biomarker and optimization of cut off points based on outcome as has been described earlier [38]. Based on XIAP expression, BCs were grouped into 2 groups based on X-tile plots: one with complete absence or reduced staining (H score = 0–85) and the other group showing over expression (H score > 85).

### Statistical analysis

Contingency table analysis and chi-square tests were used to study the relationship between clinicopathological variables and XIAP. Overall survival curves were generated using the Kaplan-Meier method, with significance evaluated using the Mantel-Cox log-rank test. The limit of significance for all analyses was defined as a *p*-value of 0.05; two-sided tests were used in all calculations. The JMP 9.0 (SAS Institute, Inc., Cary, NC) software package was used for data analyses.

### Cell culture

Breast cancer (BC) cell lines, CAL-120 (ACC 459) was obtained from DSMZ (Braunschweig, Germany). EVSAT (CSC-C0468) was purchased from Creative Bioarray (NY, USA). MCF7 (ATCC® HTB-22™) and MDA-MB-231 (ATCC® HTB-26™) were obtained from ATCC (Manassas, VA). All the cell lines were cultured in RPMI 1640 media supplemented with 10% Fetal Bovine Serum (FBS), Pen-Strep and Glutamine as described previously [30]**.** All experiments were performed using 5% FBS in RPMI 1640 media. All the cell lines were authenticated in house using short tandem repeats PCR.

### Reagents and antibodies

Embelin was purchased from Tocris Bioscience (Ellisville, MO). MTT was purchased from Sigma (St. Louis MO, MA). LY294002 and zVAD-fmk was purchased from Calbiochem (San Diego, CA, USA). XIAP antibody was purchased from BD Transduction lab (San Jose, CA, USA). Antibodies against caspase-9, caspase-3, PARP, p-AKT, p-Bad, Bcl-2, Bcl-Xl, Beta-actin, Survivin and Bid were purchased from Cell Signaling Technologies (Beverly, MA, USA). Cytochrome c and GAPDH antibodies were purchased from Santa Cruz Biotechnology, Inc. (Santa Cruz, CA, USA). cIAP-1 antibody was purchased from R&D (USA). Annexin V/PI staining kit was purchased from Molecular Probes (Eugene OR, USA).

### Cell lysis and immunoblotting

Following treatment with inhibitors or siRNA, BC cells were lysed and proteins were isolated as previously described [39]. Following protein isolation, equal amount of protein were separated by SDS-Page and immunoblotted with different antibodies. The blots were developed using enhanced chemiluminescence (ECL, Amersham, Illinois, USA) system.

### 3-(4,5-Dimethylthiazol-2-yl)-2,5-Diphenyltetrazolium bromide (MTT) assays

BC cells were plated at a density of 10^4^ cells for 24 h in 96 well plates and were treated with different doses of embelin or LY294002 for 24 h at a final volume of 200 μl. MTT assays were performed to determine cell viability using a plate reader as previously described [40]. Results depicted are from three independent experiments. *p < 0.05 and **p < 0.005.

### Cell cycle analysis and apoptosis assay

For cell cycle analysis and annexin V/PI staining for apoptosis, following treatment with 25 and 50 μM embelin, cells were harvested and washed with 1× PBS and re-suspended in either 500 μl hypotonic staining buffer for cell cycle analysis or annexin V/PI for apoptosis assay. Following incubation, cells were analyzed by flow cytometry as shown before [41].

### Assay for cytochrome c release

Following treatment with embelin for 24 h, mitochondrial free cytosolic extracts and cytosolic free mitochondrial extracts were isolated as described previously [30]. Equal amount of protein (20 μg) were separated with SDS-Page and immunoblotted with antibodies against anti-cytochrome c, GAPDH and Cox IV antibodies.

### Measurement of mitochondrial potential

Following treatment with Embelin, cells were stained with JC1 dye and incubated at 37 °C for 30 min in the dark. After incubation, cells were washed, re-suspended in PBS and analyzed by flow cytometry as described early [42].

### Gene silencing using siRNA

XIAP siRNA (Cat no. 6550 and 6446) were purchased from Cell Signaling, AKT siRNA (Cat no. SI02757244 and SI02758406) as well as Scrambled control siRNA (Cat no. 1027281) were purchased from Qiagen (Valencia, CA, USA). siRNA were transfected into breast cancer cell lines as described previously [12]. Following 48 h transfection, proteins were isolated and expression was determined by Western Blot analysis.

### Animals and xenograft study

Female nude mice were chosen for these experiments and mice were injected with MDA-MB-231 cells (10 million per animal). Following one week of injection, the animals were randomly assigned into three groups. The first groups were not treated and only vehicle (DMSO) was injected while the other two groups were treated with 10 and 20 mg/kg embelin, injected intra-peritoneally, twice weekly for 4 weeks respectively. In the second set of experiments, the female mice were divided into four groups, the first group received DMSO alone, while the second and third received embelin (10 mg/kg) and LY294002 (10 mg/kg). The fourth group received a combination of embelin and LY294002, injected simultaneously. During the study, the weight and tumor volume of each mouse was monitored weekly. After 4 weeks of treatment, mice were sacrificed and individual tumors were weighed, and then snap frozen in liquid nitrogen for storage.

## Results

### Determination of XIAP expression by IHC and correlation with clinical data and molecular markers

**Fig. 1:**
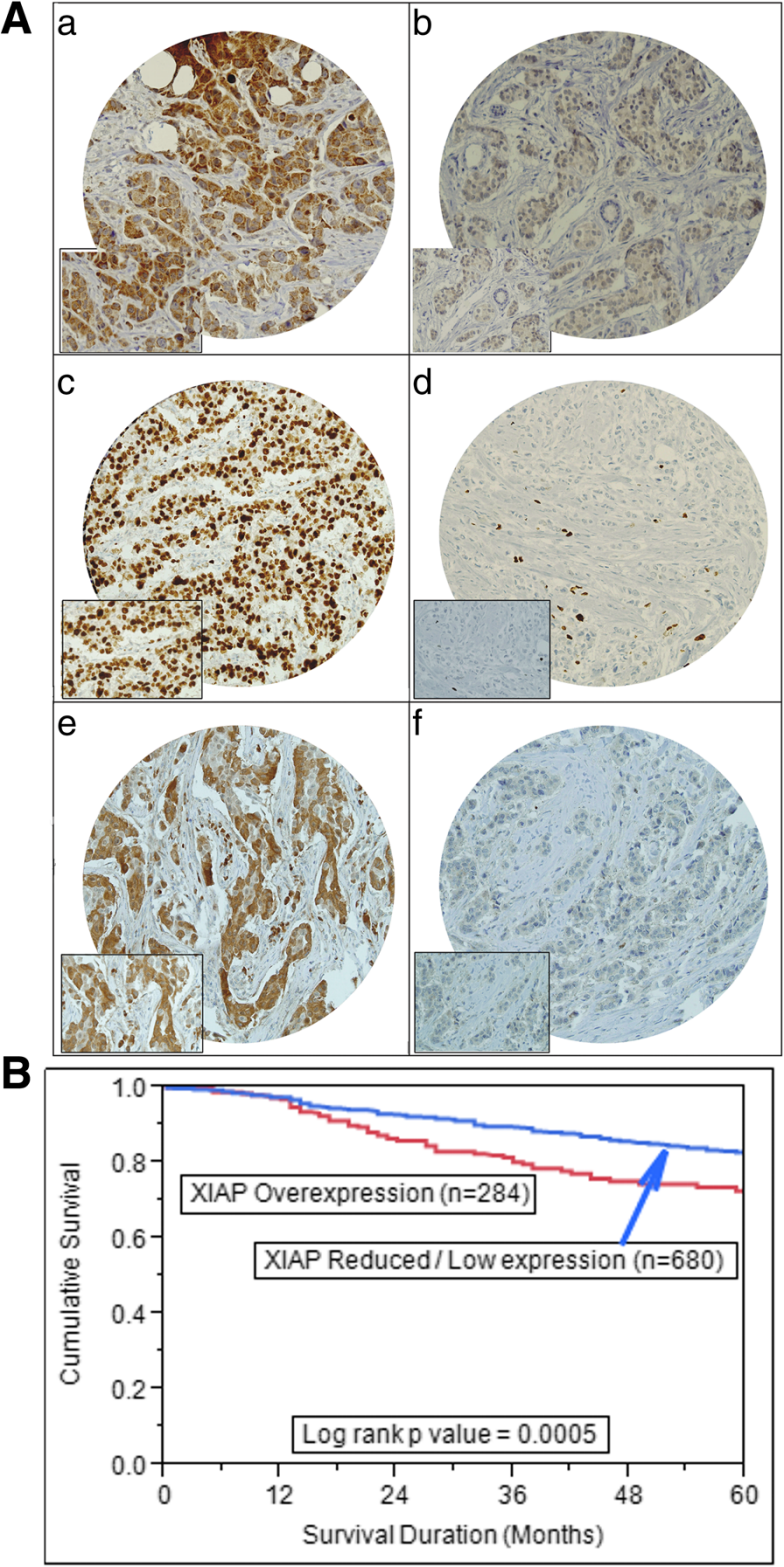
(A) Tissue microarray based Immunohistochemical analysis in breast cancer patients. (a) Breast cancer TMA spot showing XIAP overexpression as compared to another breast cancer spot showing low XIAP expression (b). (c) Breast cancer tissue array spots showing high proliferative index of Ki-67 as compared to another breast cancer spot showing negligible expression of Ki67 (d). (e) Breast cancer TMA spot showing high activation of AKT as compared to another spot showing low activation level of AKT (f). 20 X/0.70 objective on an Olympus BX 51 microscope. (Olympus America Inc. Center Valley, PA, USA) with the inset showing a 40X 0.85 aperture magnified view of the same TMA spot. (B) Kaplan-Meier survival analysis for the prognostic significance of XIAP expression in breast cancer. Breast cancer patients with overexpression of XIAP had poor overall survival of 71.2 months as compared 82.8 months for patients having low expression of XIAP (*p* = 0.0005)

To identify the role of XIAP in the pathogenesis of breast cancer, we analyzed expression of XIAP by IHC on a TMA format on a large cohort of BC samples collected at KFSHRC from 1990 to 2011. Our data showed that 29.5% (284/964) BC samples had over-expression of XIAP (Table 1). Clinically, XIAP over-expression was significantly associated with tumor size (*p* = 0.0044), extra-nodal extension (*p* = 0.0041), poorly differentiated tumor (*p* < 0.0001), triple negative breast cancer (0.0019) and infiltrative ductal carcinoma subtype (*p* = 0.002). At the molecular level, XIAP over-expression significantly associated with proliferative marker; Ki67 (*p* < 0.0001), PARP (*p* < 0.0001) and p-AKT (*p* < 0.0001) (Table 1 and Fig. 1a). Finally, XIAP over-expression led to a poor overall survival of 71.8% as compared to 82.8% (*p* = 0.0005) (Fig. 1b) and was found to be an independent poor prognostic marker in multivariate analysis (Additional file 2: Table S2).

**Table 1:** Correlation of XIAP with clinico-pathological parameters in Breast Cancer

	Total		XIAP Over-expression		XIAP Low-expression		*P* value
	N	%	N	%	N	%	
Total Number of Cases	964		284	29.5	680	70.5	
Age Groups							
< 50	306	31.7	87	28.4	219	71.6	0.6320
> 50	658	68.3	197	29.9	461	70.1	
Tumor size^a^							
≤ 2 cm	208	22.1	46	22.1	162	77.9	0.0044
> 2 cm	731	77.9	236	32.1	498	67.9	
Lymph Nodes involvement^a^							
Negative	300	33.3	81	27.0	219	73.0	0.3914
Positive	602	66.7	179	29.7	423	70.3	
Metastasis^a^							
M0	776	89.8	225	29.0	551	71.0	0.1587
M1	88	10.2	32	36.4	56	63.6	
Tumor Stage^a^							
I	76	9.1	19	25.0	57	75.0	0.4453
II	366	43.7	107	29.2	259	70.8	
III	307	36.7	91	29.6	216	70.4	
IV	88	10.5	32	36.4	56	63.6	
Extra Nodal Ext.^a^							
Present	262	33.2	92	35.1	170	64.9	0.0041
Absent	527	66.8	133	25.2	394	74.8	
LVI^a^							
Present	350	41.0	110	31.4	240	68.6	0.1411
Absent	504	59.0	135	26.8	369	73.2	
Histological Grade ^a^							
Well differentiated	72	7.6	10	13.9	62	86.1	<0.0001
Moderately differentiated	489	51.3	123	25.1	366	74.9	
Poorly differentiated	393	41.2	150	38.2	243	61.8	
Histology^a^							
Infiltrating Ductal Carcinoma	878	93.7	272	31.0	606	69.0	0.0002
Infiltrating Lobular	43	4.6	3	7.0	40	93.0	
Mucinous Ca	16	1.7	2	12.5	14	87.5	
Triple Negative^a^							
No	815	84.9	225	27.6	590	72.4	0.0019
Yes	145	15.1	59	40.7	86	59.3	
Ki-67 IHC^a^							
High	610	64.3	214	35.1	396	64.9	<0.0001
Low	339	35.7	66	19.5	273	80.5	
PARP^a^							
High	433	45.2	159	36.7	274	63.3	<0.0001
Low	525	54.8	125	23.8	400	76.2	
phos_AKT (473)^a^							
Negative	728	77.4	181	24.9	547	75.1	<0.0001
Positive	212	22.6	100	47.2	112	52.8	
Survival							
OS 5 Years				71.8		82.8	0.0005

^a^Data was not available (NA) for some cases: Tumor size (NA = 25), Lymph nodes (NA = 62), Metastasis (NA = 100), Tumor Stage (NA = 127), Extra Nodal Ext. (NA = 175), LVI (NA = 110), Histological Grade (NA = 10), Histology (NA = 27), Triple Negative (NA = 04), Ki-67 (NA = 15), PARP (NA = 06), & phos. AKT(473) (NA = 24)

### Down-regulation of XIAP using embelin inhibited cell viability and induced apoptosis in BC cells

**Fig. 2:**
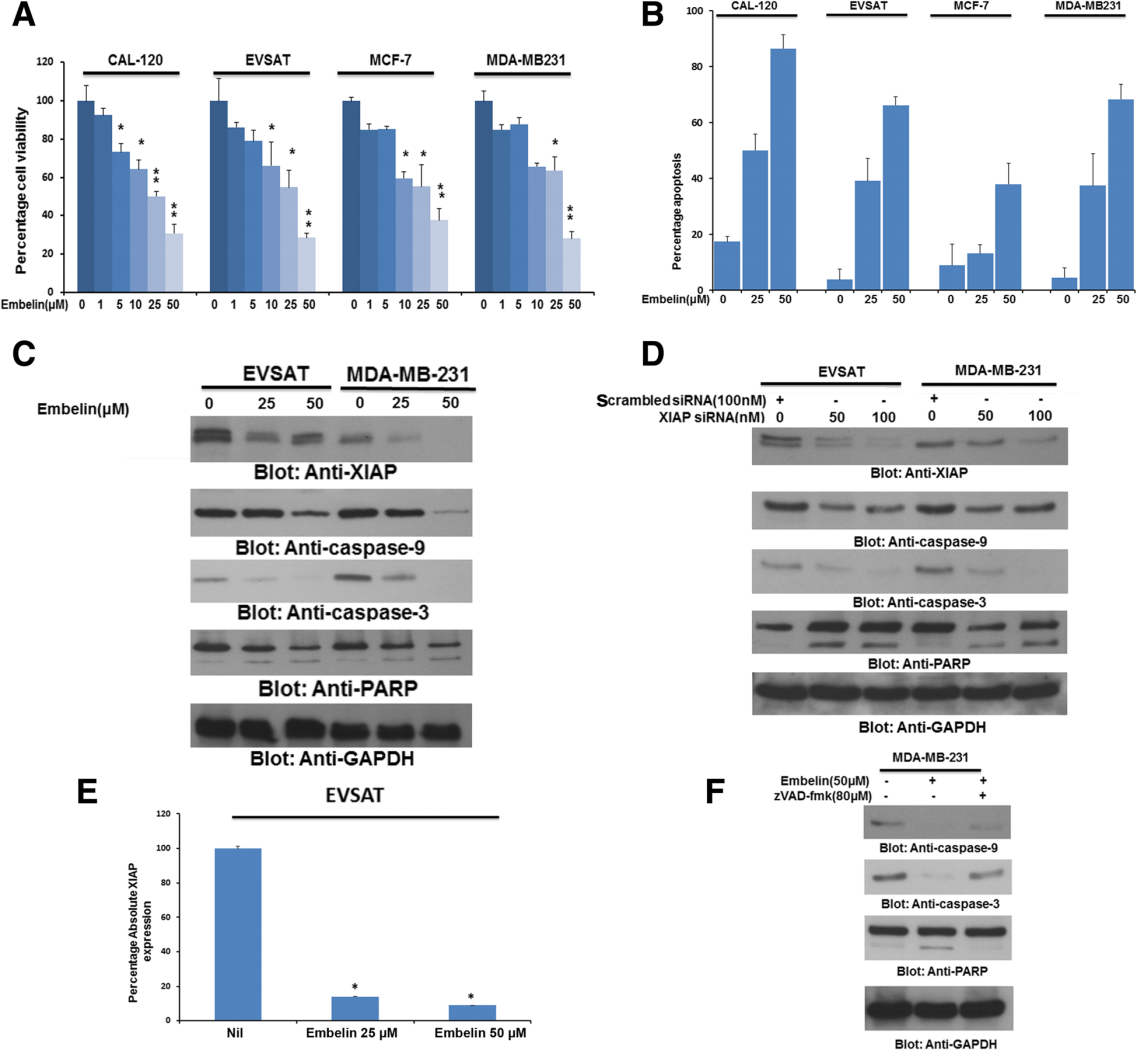
(**a**) Embelin inhibits cell viability in BC cells. (**a**) Breast cancer cells; CAL-120, EVSAT, MCF-7 and MDA-MB-231cells were treated with increasing doses of embelin ranging between 0 and 50 μM concentration. Cell viability assays were performed using MTT as described in Materials and methods. The graph displays the mean +/− SD (standard deviation) of three independent experiments with replicates of six wells for all the doses and vehicle control for each experiment * *p* < 0.01 and ** *p* < 0.001, statistically significant (Students *t*-test). (**b**) Embelin treatment induces apoptosis in BC cells. BC cells were treated with increasing doses of embelin for 24 h and cells were analysed for apoptosis after staining with annexin V/PI dual staining by flow cytometry. (**c**) Embelin inhibits expression of XIAP and induces cleavage of caspases-9, −3 and PARP in BC cells. EVSAT and MDA-MB-231 cells were treated with 25 and 50 μM embelin for 24 h. After cell lysis, equal amounts of proteins were separated on SDS-PAGE, and immunoblotted with antibodies against XIAP, caspase-9, caspase-3 and GAPDH as indicated. (**d**) XIAP siRNA transfection down-regulates XIAP expression and activates caspases in BC cells. EVSAT and MDA-MB-231 cells were transfected with either scrambled siRNA or specific siRNA against XIAP for 48 h. Following transfection, proteins were isolated and probed with antibodies against XIAP, caspase-9, caspase-3, PARP and GAPDH. (**e**) Embelin down-regulates XIAP transcript in BC cells. EVSAT cells were treated with 25 and 50 μM Embelin for 24 h and RNA were isolated, reverse transcribed into cDNA. Serial dilutions of untreated EVSAT cells cDNA were used to generate a standard curve for GAPDH and XIAP expression. Following treatment, quantitative RT-PCR was performed on cDNA of PTC cells treated with and without 25 and 50 μM Embelin for the expression of XIAP and GAPDH. Absolute qRT-PCR analysis was performed using ABI-7900HT Fast Real-Time PCR system. The results were plotted on a bar graph and standard deviation calculated. Three replicates for each sample were used. (**f**) Embelin-induced apoptosis in BC cells is caspase dependent. MDA-MB-231 cells were either pre-treated with universal caspase inhibitor, zVAD-fmk for 3 h followed by treatment with embelin for 24 h. Proteins were isolated and probed with antibodies against caspase-9, caspase-3, PARP and GAPDH

Our clinical data showed that XIAP over-expression was associated with a significant 5 year poor survival of 71.8% (*p* = 0.005) (Table 1). Therefore, we wanted to investigate whether XIAP could be targeted using a specific XIAP inhibitor, embelin [28] to inhibit cell growth and induce apoptosis in BC cells. Therefore, we treated four BC cell lines; CAL-120, EVSAT, MCF-7 and MDA-MB-231 with increasing doses of Embelin for 24 h to assess cell viability using MTT assay. As shown in Fig. 2a, Embelin inhibited cell viability in all the four cell lines that expressed XIAP in a dose dependent manner. Next, to determine whether embelin induced cell inhibition was due to apoptosis, we treated BC cells with increasing doses of embelin for 24 h and analyzed the cells for apoptosis after dual staining with annexin V/PI by flow cytometry. As shown in Fig. 2b, all the four BC cell lines underwent apoptosis at increasing doses however the IC50 of all four cell lines ranged between 25 and 50 μM concentration of embelin and therefore, the rest of the experiments were performed at 25 and 50 μM only. Once, it was ascertained that the BC cells were undergoing apoptosis following embelin treatment, we wanted to determine whether embelin treatment of BC cells down-regulated expression of XIAP and induced caspase dependent apoptosis. Therefore we chose two cell lines; EVSAT and MDA-MB-231 and treated them with 25 and 50 μM embelin for 24 h. Following treatment, proteins were isolated and probed with antibodies against XIAP, caspases-9 and -3, PARP and GAPDH. Our data showed that embelin treatment caused down-regulation of XIAP expression and cleavage of caspases-9 and -3 in both the cells as demonstrated by decreased intensity of pro-bands. In addition, embelin treatment also induced cleavage of PARP, a protein that needs to be cleaved for efficient apoptosis to occur [43, 44] (Fig. 2c). To confirm these findings, we also transfected EVSAT and MDA-MB-231 with either non-specific scrambled siRNA or siRNA targeted against XIAP and assessed the protein expression following transfection by immunoblotting. As shown in Fig. 2d, we found similar results with down-regulation of XIAP thereby confirming the role of embelin in inducing caspase-dependent apoptosis in BC cells. XIAP down-regulation was also confirmed using another XIAP siRNA (Data not shown). Embelin treatment also transcriptionally down-regulated expression of XIAP in EVSAT cells as detected by real-time RT-PCR (Fig. 2e). Furthermore, we also pre-treated MDA-MB-231 cells with a universal caspase-inhibitor, zVAD-fmk for three hours followed by treatment with 50 μM embelin for 24 h. As shown in Fig. 2f, zVAD-fmk pre-treatment restored expression of caspases-9, −3 and inhibited PARP breakdown in BC cells. This data confirmed that embelin-induced apoptosis is caspase dependent.

### Embelin treatment activated mitochondrial apoptotic pathway via in-activation of AKT in BC cells

**Fig. 3:**
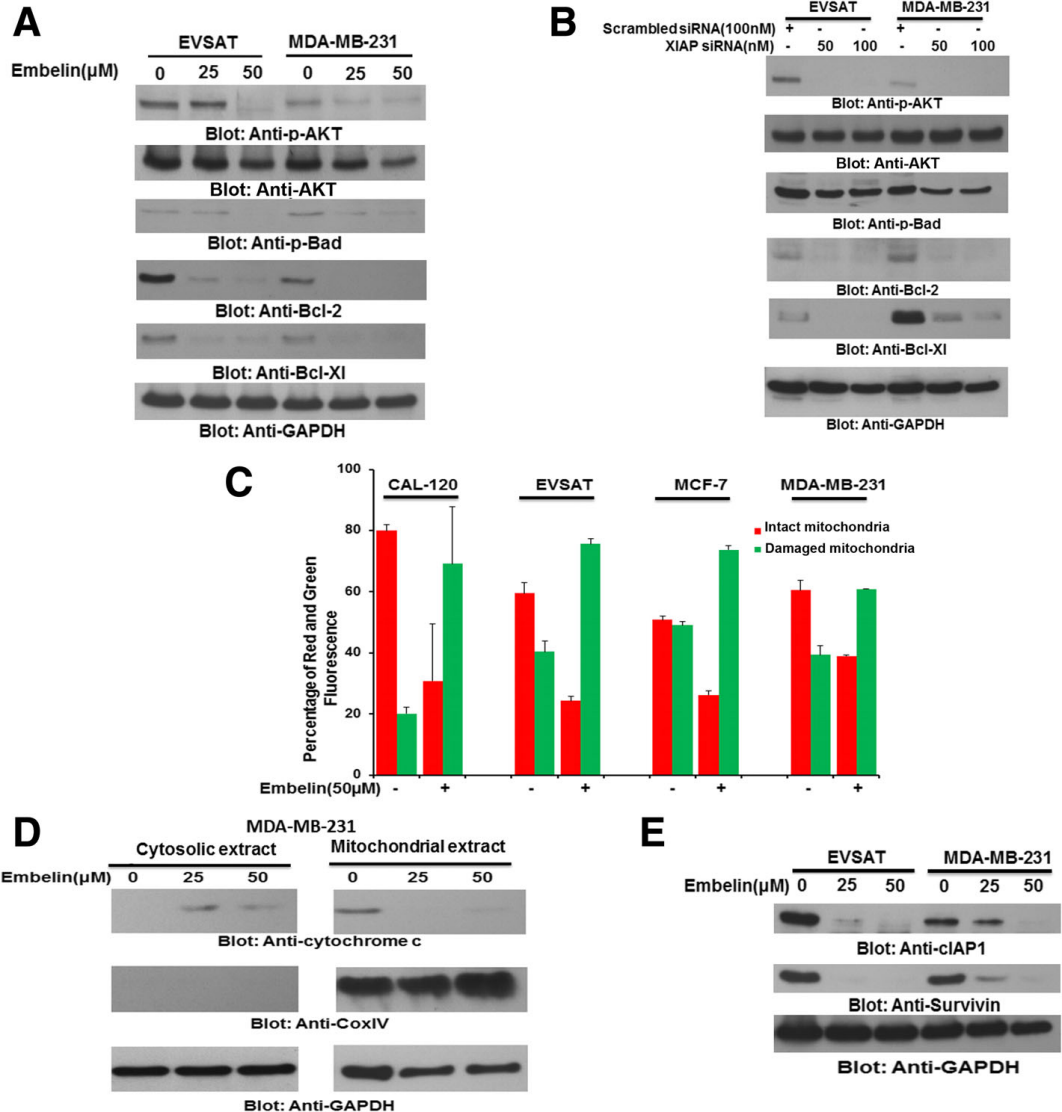
Embelin causes activation of mitochondrial apoptotic pathway via inactivation of AKT in BC cells. (**a**) Embelin treatment causes inactivation of AKT, Bad and down-regulation of Bcl-2 and Bcl-Xl in BC cells. EVSAT and MDA-MB-231 cells were treated with 25 and 50 μM embelin for 24 h and proteins were isolated, separated on SDS-Page and probed with antibodies against p-AKT, AKT, p-Bad, Bcl-2, Bcl-Xl and GAPDH. (**b**) Embelin induced inactivation of AKT and down-stream targets are confirmed by siRNA transfection against XIAP. EVSAT and MDA-MB-231 cells were transfected with either non-specific scrambled siRNA or specific siRNA targeted against XIAP for 48 h. Following transfection, proteins were isolated and probed with antibodies against p-AKT, AKT, p-Bad, Bcl-2, Bcl-Xl and GAPDH. (**c**) Change in mitochondrial membrane potential determined by JC1 staining following Embelin treatment in BC cells. BC cells were treated with 50 μM of embelin for 24 h and following treatment, cells were stained with JC1 and analysed for red stained cells (intact mitochondria) and green stained cells (damaged mitochondria) by flow cytometry. (**d**) Embelin-induced release of cytochrome c in BC cells. MDA-MB-231 cells were treated with 25 and 50 μM Embelin for 24 h. Following treatment, mitochondrial free cytosolic extracts as well as mitochondrial extracts were isolated and probed with antibodies against cytochrome c, Cox IV and GAPDH. (**e**) Embelin treatment also down-regulates expression of IAPs in BC cells. EVSAT and MDA-MB-231 cells were treated with embelin for 24 h and proteins were probed with antibodies against cIAP1 and Survivin. GAPDH was used as a loading control

Our clinical data on the cohort of BC samples showed a significant association between XIAP expression and activated AKT. In addition, we and others have also shown that XIAP expression and activated AKT are closely associated in rendering a cancer cell resistant and aggressive [14, 45]. Therefore, we sought to determine whether this association was present in BC cells and whether down-stream target of AKT, p-Bad also be inactivated following treatment of Embelin leading to activation of mitochondrial apoptotic pathway. EVSAT and MDA-MB-231 cells were treated with 25 and 50 μM embelin for 24 h and proteins were immunoblotted with antibody against p-AKT and p-Bad. As shown in Fig. 3a, embelin treatment inactivated AKT and Bad in both the cell lines tested. For mitochondrial apoptotic pathway to be activated, two anti-apoptotic members of the Bcl-2 family of proteins, Bcl-2 and Bcl-Xl, need to be down-regulated for the apoptotic signal to reach the mitochondria [46]. Our data showed that in addition to inactivation of p-AKT and p-Bad, there was also down-regulation of Bcl-2 and Bcl-Xl following treatment with embelin in BC cell lines (Fig. 3a). Similar results were obtained following transfection with specific siRNA targeting XIAP gene confirming specificity as well as negating off-target effects of embelin in BC cells (Fig. 3b). Once the apoptotic signal reaches the mitochondria, it causes changes in the mitochondrial membrane potential due to damage to the mitochondrial membrane causing release of cytochrome c into cytosol. To assess changes in mitochondrial membrane potential, all four BC cell lines following treatment with embelin were stained with JC1 dye to determine the mitochondrial membrane potential [47]. As shown in Fig. 3c, there was a shift of red stained normal cells towards green stained damaged cells following treatment with embelin confirming change in mitochondrial membrane potential. We also found that embelin treatment of MDA-MB-231 cells led to release of cytochrome c into cytosol from the mitochondria (Fig. 3d). The immunoblots were also probed with antibody against Cox IV to confirm fractionation of samples into pure mitochondrial and mitochondrial–free cytosolic extracts and GAPDH to denote equal loading. Finally, in addition of XIAP, we also investigated down-regulation of other members of IAP family members, cIAP1 and survivin, following embelin treatment and found that both; cIAP1 and Survivin were down-regulated following embelin treatment (Fig. 3e).

### XIAP treatment regressed BC cell xenografts in vivo

Once we confirmed the efficacy of embelin in inducing apoptosis via down-regulation of XIAP in vitro, we wanted to assess the response of BC cell xenografts in vivo on nude mice. Female nude mice (*n* = 12) were inoculated with MDA-MB-231 cells (10 × 10^6^) in the right abdominal quadrant for 1 week and then mice were divided into three groups. The first group was treated with DMSO alone (control vehicle) while the other two groups were treated with 10 and 20 mg/kg body weight of embelin, injected twice weekly intraperitoneally for 4 weeks. The tumor volume was measured weekly and after 4 weeks of treatment, the animals were sacrificed, the tumors were weighed and proteins were isolated. Our data showed a significant decrease in volume at 20 mg/kg body treatment with embelin from second week onwards (Additional file 3: Figure S1A). The tumor weight also decreased in xenografts treated with 10 and 20 mg/kg embelin. However, there was a significant difference between vehicle and 20 mg/kg embelin treated xenografts (Additional file 3: Figure S1B). On naked eye examination, there was a visible decrease in the size of the xenografts treated with 10 and 20 mg/kg Embelin when compared to vehicle treated xenografts (Additional file 3: Figure S1C). Finally, when isolated proteins from the three groups of xenografts were immunoblotted to confirm our in vitro findings, there was down-regulation of XIAP, Bcl-2 and Bcl-Xl, inactivation of AKT and breakdown of caspase-3 as shown in Additional file 3: Figure S1D. These results confirmed that embelin was regressing MDA-MB-231 xenografts via down-regulation of XIAP.

### Synergistic apoptotic response of BC cells to combination of XIAP and PI3-kinase/AKT inhibitors

**Fig. 4:**
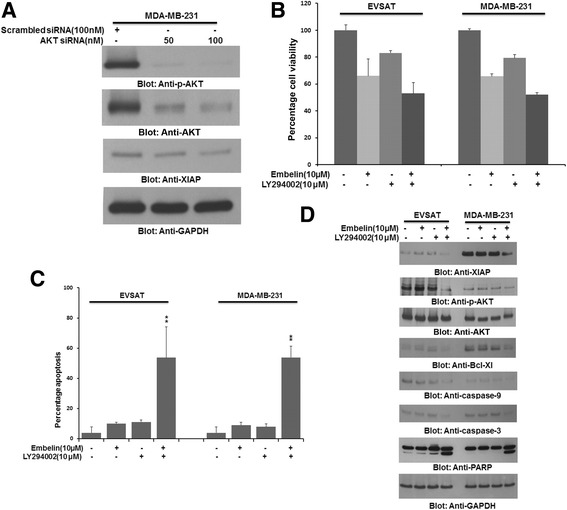
Combination treatment with sub-optimal doses of embelin and LY294002 synergistically induces apoptosis in BC cells. (**a**) AKT siRNA down-regulates expression of XIAP in BC cells. MDA-MB-231 cells were transfected with siRNA targeted against AKT and proteins were isolated and probed with antibodies against p-AKT, AKT, XIAP and GAPDH. (**b** and **c**) Combination of embelin and LY294002 at sub-optimal doses synergistically inhibits cell viability and induces apoptosis in BC cells. EVSAT and MDA-MB-231 cells were treated with either alone or in combination of embelin (10 μM) and LY294002 (10 μM) for 24 h. Following 24 h treatment, cells were analysed for cell viability by MTT assays (**b**) and apoptosis after staining the cells with annexin V/PI by flow cytometry (**c**). (**d**) Combination treatment causes inactivation of AKT, down-regulation of XIAP and caspase-dependent apoptosis in BC cells. EVSAT and MDA-MB-231 cells were treated with combination of sub-toxic doses of embelin and LY294002 for 24 h. Following incubation, proteins were isolated and probed with antibodies against XIAP, p-AKT, AKT, Bcl-Xl, caspase-9, caspase-3, PARP and GAPDH

Embelin treatment of BC cells not only down-regulated XIAP expression but also inactivated AKT (Fig. 2). In addition, we also found significant association between XIAP over-expression and p-AKT in BC samples (Table 1). We were therefore interested to determine whether AKT down-regulation could inhibit XIAP expression in BC cells. MDA-MB-231 cells were transfected with specific siRNA against AKT and cells were evaluated for XIAP expression by immunoblotting. Our data showed that in addition to inactivation of AKT following siRNA transfection, XIAP was also downregulated in MDA-MB-231 cells (Fig. 4a). This data suggested that a feedback mechanism was active between XIAP and AKT in BC cells. Our data is in concordance and supports previously published studies [48, 49]. Next, we sought to determine whether combination of XIAP inhibitor and PI3-kinase/AKT inhibitor, LY294002 could synergistically inhibit cell viability and induce apoptosis in BC cells. We initially conducted multiple experiments with varying doses of embelin and LY294002 in different combinations to determine the optimal dose of combination that could synergistically induce apoptosis in BC cells. Using Chou and Talalay method and Calcusyn software [50], we found that 10 μM embelin and 10 μM LY294002 had a combination index of 0.447 in EVSAT cell line and 0.368 in MDA-MB-231 cell line suggesting a synergistic apoptotic response (Additional file 4: Table S3 and Additional file 5: Figure S2). Using these doses, we treated BC cells for 24 h and found that combination of embelin and LY294002 inhibited cell viability and induced significant apoptosis as compared to treatment alone with single inhibitor (Fig. 4b and c). We were at the same time also interested in identifying the proteins involved in apoptosis with combination of the two inhibitors. After treatment with either embelin or LY294002 alone or in combination for 24 h, proteins were isolated and immunoblotted with different antibodies. In addition to down-regulation of XIAP and inactivation of AKT, combination of sub-toxic doses of embelin and LY294002 down-regulated expression of Bcl-Xl and caused cleavage of caspases-9, −3 and PARP (Fig. 4d). These data clearly suggested that combination of embelin and LY294002 at sub-toxic doses induced efficient caspase-dependent apoptosis in BC cells via down-regulation of XIAP and inactivation of AKT.

### Combination of XIAP and LY294002 synergistically regress BC cell xenografts in vivo

**Fig. 5:**
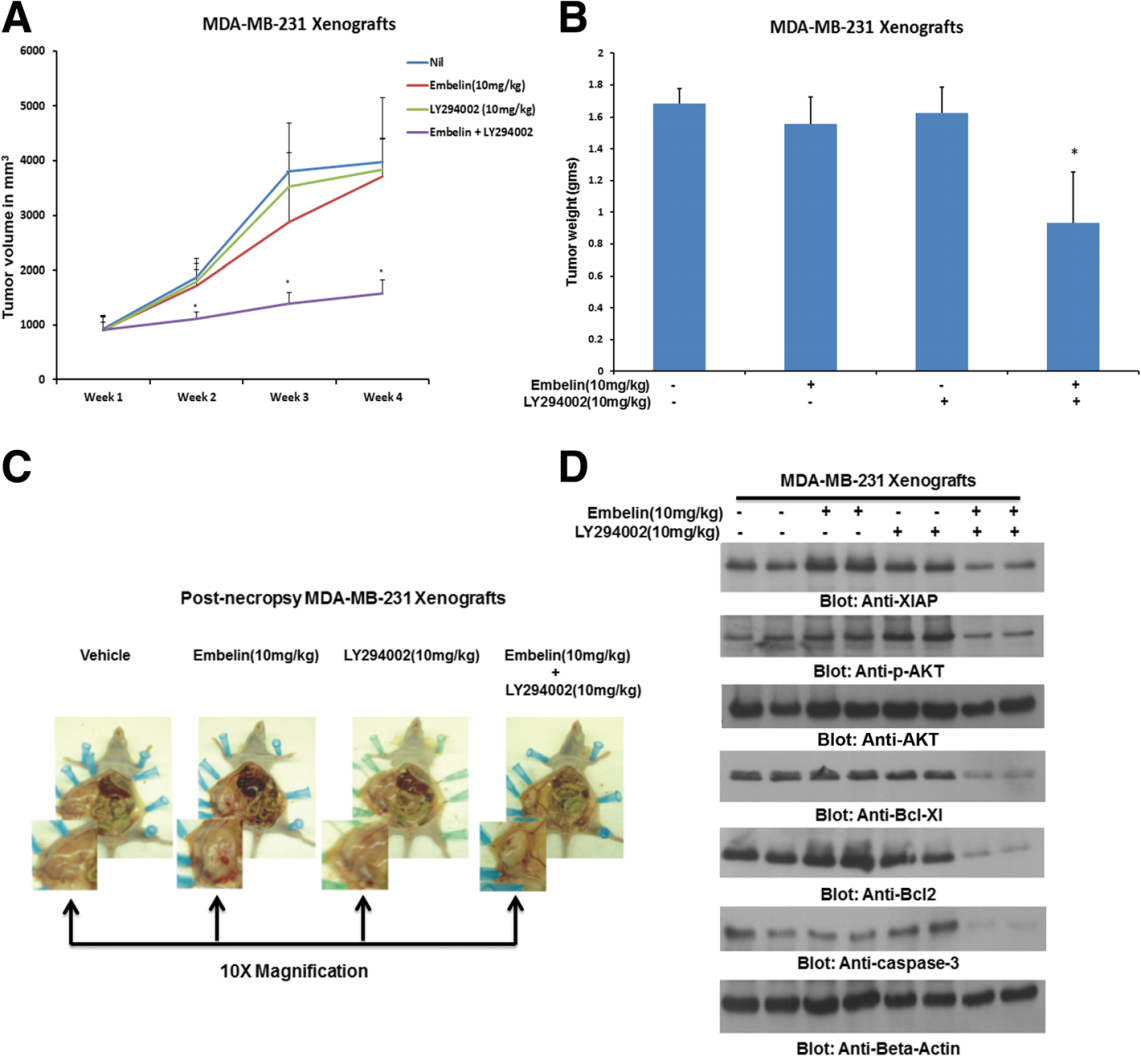
Embelin/LY294002 combination inhibits growth of MDA-MB-231 Xenografts. Female nude mice at 6 weeks of age were injected S.C. with 10 million MDA-MB-231 cells. After one week, mice were divided into four groups; the first group only received DMSO vehicle alone; the second and third group were treated with either embelin (10 mg/kg) or LY294002 (10 mg/kg) and the fourth group were treated with combination of embelin and LY294002. (**a**) Volume of each tumour was measured every week. The average (*n* = 4) tumour volume of mice was calculated, * *p* < 0.05 inhibition of MDA-MB-231 tumour growth by combination of embelin and LY294002. (**b**) After 4 weeks of treatment, mice were sacrificed and tumour weights were measured,*p < 0.05 compared to vehicle-treated mice by Student’s t-test. (**c**) Representative tumour images of vehicle, embelin, LY294002 and combination of embelin and LY294002 treated mice. (**d**) Whole cell lysate from mice treated with different inhibitors were isolated and 10 μg protein were separated by SDS-PAGE, transferred to PVDF membrane, and immunoblotted with antibodies against XIAP, p-AKT, AKT, Bcl-Xl, Bcl-2, caspase-3 and Beta-actin

Once in vitro data confirmed that combination of sub-toxic doses of embelin and LY294002 could induce caspase-dependent apoptosis via down-regulation of XIAP and inactivation of p-AKT, we wanted to ascertain whether this combination was viable in vivo on BC xenografts. We therefore injected 10 million MDA-MB-231 cells in female nude mice and after 1 week of inoculation, divided the animals into four groups. One group remained untreated while the other three groups were treated with either 10 mg/kg embelin or 10 mg/kg LY294002 alone or in combination for 4 weeks. After 4 weeks, the animals were sacrificed and the proteins were isolated from tumor tissue. There was a significant reduction in tumor volume and tumor weight in animals treated with combination of embelin and LY294002 as compared to treatment alone (Fig. 5a–c). When protein expression was assessed in tumor samples by immunoblotting, there was down-regulation of XIAP and inactivation of AKT and subsequent down-stream targets thereby suggesting that tumor regression in xenografts were following the same pattern as the in vitro studies in BC cell lines (Fig. 5d).

## Discussion

Breast cancer continues to be a debilitating dilemma for women suffering from this disease with regards to mortality and morbidity all over the world. For these reasons, researchers all over the world are actively trying to identify pre-existing or new molecular targets that can be targeted for improving the outcome, in terms of progression as well as overall survival of breast cancer patients. In our search for druggable molecular targets, we found that XIAP was over-expressed in 29.5% of breast cancer and was significantly associated with adverse clinical parameters such as large tumor size, extra-nodal extension and high tumor grade of breast cancer. In addition, XIAP over-expression was found to be associated with poor survival and was found to be an independent poor prognostic marker in multi-variate analysis. Even though XIAP over-expression has been shown to have poor survival in breast cancer in other population, however, there is limited information on the role of XIAP in Middle Eastern populations [51, 52]. This data is in concordance with data of breast cancer in other population and identifies XIAP as a poor prognostic marker for breast cancer in Middle Eastern population. While XIAP over-expression was found to have poor overall survival and can be used as a viable prognostic marker in breast cancer, we were also interested in utilizing XIAP expression as a therapeutic target in breast cancer. We have previously shown that XIAP expression can be successfully targeted in DLBCL and PTC leading to inhibition of cell viability via inducing caspase-dependent apoptosis [12, 14]. Using embelin, a specific inhibitor of XIAP that acts by disrupting the interaction between BIR3 domain of XIAP with caspase-9, we found that there was inhibition of cell viability and caspase-dependent apoptosis at doses of 25 and 50 μM concentration. While the doses of embelin were high, we also found that these doses did not induce apoptosis in normal peripheral blood mono-nuclear cells (PBMNC) (Data not shown). These results highlight the importance of targeting XIAP in a subset of breast cancer with over-expression of XIAP.

Monotherapy using small molecular inhibitors or antibodies used for treatment of cancer have had their share of success and failure where some inhibitors/antibodies have done well when used alone [53], however, many experimental inhibitors/antibodies have failed due to either acquired resistance to therapy or increased toxicities following initial success [54, 55]. On the other hand, these experimental agents have fared much better when used in combination with other inhibitors or chemotherapeutic agents [56, 57]. An important advantage of using combination therapy is that usually sub-optimal doses are required to induce a synergistic response thereby decreasing the chances of toxicities that are usually present with using high doses with the same inhibitors alone. As our clinical data showed a significant association between XIAP over-expression and activated AKT and it has also been shown that XIAP and AKT are inter-linked in various cancer [12, 30], we speculated that combination of PI3-kinase inhibitor with embelin would be a suitable strategy to treat breast cancer cells. Our in vitro and in vivo data clearly indicated the utility of targeting a subset of breast cancer with over-expression of XIAP and activated AKT to successfully inhibit cell growth and induce apoptosis.

## Conclusions

We found that XIAP was over-expressed in one third of our cohort of breast cancer samples and elicited a poor survival. Targeting XIAP both alone and in combination with LY294002 induced apoptosis in vitro and caused regression of breast cancer xenografts in vivo suggesting a role of XIAP in breast cancer tumorigenesis and at the same time, identifying XIAP as a potential therapeutic target. Finally, using these strategies in clinical setting can improve the management of breast cancer in the future. However, further in-depth studies are required to study the efficiency and associated toxicities of these agents before they can be fully utilized for the management of sub-group of breast cancer with XIAP over-expression.

## Additional files

Additional file 1: **Table S1.** Details of primary antibodies, dilutions and supplier. (DOCX 14 kb)

Additional file 2: **Table S2.** Univariate and Multivariate analysis of XIAP using Cox Proportional Hazard Model. (DOCX 20 kb)

Additional file 3: **Figure S1.** Inhibition of PTC cell tumor-xenografts growth by embelin. Female nude mice at 6 weeks of age were injected subcutaneously with ten million MDA-MB-231 cells. After one week, the animals were randomly divided into three groups. The first groups were not treated and only vehicle (DMSO) was injected while the other two groups were treated 10 and 20 mg/kg embelin, injected intra-peritoneally, twice weekly for 4 weeks respectively. (**A**) The volume of each tumor was measured every week. The average (*n* = 4) tumor volume in each group of mice was calculated, * *p* < 0.05. (**B**) After 4 weeks treatment, mice were sacrificed and mean tumor weight (±SD) was calculated in each group. (**C**) Representative tumor images of each group of mice after necropsy. Inset showing 10X magnification. (**D**) Whole-cell homogenates from mice injected with TPC1cells were immuno-blotted with antibodies against XIAP, p-AKT, AKT, Bcl-Xl, Bcl-2 caspase 3 and beta-actin. (TIFF 9495 kb)

Additional file 4: **Table S3.** Combination index calculation using Chou and Talalay method in BC cell lines. (DOCX 17 kb)

Additional file 5: **Figure S2.** Synergistic apoptotic response of embelin and LY294002 in BC cells. EVSAT and MDA-MB-231 cells were treated with various combinations of embelin and LY294002 for 24 h and dose effect (A and B) and Fractional effect (C and D) graphs were generated using Calcusyn software. Apoptotic response analysis was done as mean ± SD values normalized to control. Combination indices were calculated using Chou and Talalay methodology. (TIFF 1124 kb)

## Abbreviations

BC: Breast cancer
cIAP1: Cellular inhibitor of apoptosis
JC1: Tetraethylbenzimidazolylcarbocyanine iodide
MTT: (3-(4,5-Dimethylthiazol-2-yl)-2,5-Diphenyltetrazolium Bromide)
XIAP: X-Linked inhibitor of apoptosis
