# Supplementary material for: XIAP over-expression is an independent poor prognostic marker in Middle Eastern breast cancer and can be targeted to induce efficient apoptosis
Source: BMC Cancer. 2017 Sep 11;17:640. doi: 10.1186/s12885-017-3627-4 (PMC5594504; doi:10.1186/s12885-017-3627-4)
Supplement: Supplementary file 1 — Table S1. Details of primary antibodies, dilutions and supplier. (DOCX 14 kb) [file 12885_2017_3627_MOESM1_ESM.docx]

| **Antibody** | **Clone** | **Company** | **Dilution** | **Retrieval** | **Detection** |
| --- | --- | --- | --- | --- | --- |
| Ki-67 | MIB-1 | DAKO | 1:500 | pH9, pressure cooker | Envision+ |
| p-AKT(Ser473) | D9E | Cell Signaling | 1:20 | pH9, pressure cooker | Envision+ |
| XIAP | 48 | BD | 1:400 | pH9, pressure cooker | Envision+ |
| BCL-XL | 54H6 | Cell Signaling | 1:800 | pH9, pressure cooker | Envision+ |
| PARP | 46D11 | Cell Signaling | 1:500 | pH9, pressure cooker | Envision+ |
|  |  |  |  |  |  |

**Supplementary Table 1: Details of primary antibodies, dilutions and supplier.**
