# Supplementary material for: XIAP over-expression is an independent poor prognostic marker in Middle Eastern breast cancer and can be targeted to induce efficient apoptosis
Source: BMC Cancer. 2017 Sep 11;17:640. doi: 10.1186/s12885-017-3627-4 (PMC5594504; doi:10.1186/s12885-017-3627-4)
Supplement: Supplementary file 2 — Table S2. Univariate and Multivariate analysis of XIAP using Cox Proportional Hazard Model. (DOCX 20 kb) [file 12885_2017_3627_MOESM2_ESM.docx]

**Supplement Table 2: Univariate and Multivariate analysis of XIAP using Cox Proportional Hazard Model**

| Clinical Parameters | **UNIVARIATE** | | **MULTIVARIATE** | |
| --- | --- | --- | --- | --- |
|  | Risk Ratio (95% CI) | p value | Risk Ratio (95% CI) | p value |
| **Age**  Above>50 | 0.93 (0.67-1.28) | 0.6678 | 0.80 (0.55-1.15) | 0.2296 |
| **Stage**  IV | 7.04 (4.96-9.85) | **< 0.0001** | 5.63 (3.77-8.23) | **< 0.0001** |
| **Grade**  Poorly Diff. | 1.56 (1.16-2.10) | **0.0034** | 1.44 (1.03-2.03) | **0.0364** |
| **Histology**  IDC | 0.45 (0.20-0.86) | **0.0132** | 0.40 (0.12-0.95) | **0.0373** |
| **Nodal Involvement** | 2.91 (1.94-4.52) | **< 0.0001** | 2.77 (1.80-4.46) | **< 0.0001** |
| **XIAP** | 1.71 (1.25-2.33) | **0.0009** | 1.59 (1.12-2.24) | **0.0099** |
