# Supplementary figures and images for: XIAP over-expression is an independent poor prognostic marker in Middle Eastern breast cancer and can be targeted to induce efficient apoptosis
Source: BMC Cancer. 2017 Sep 11;17:640. doi: 10.1186/s12885-017-3627-4 (PMC5594504; doi:10.1186/s12885-017-3627-4)

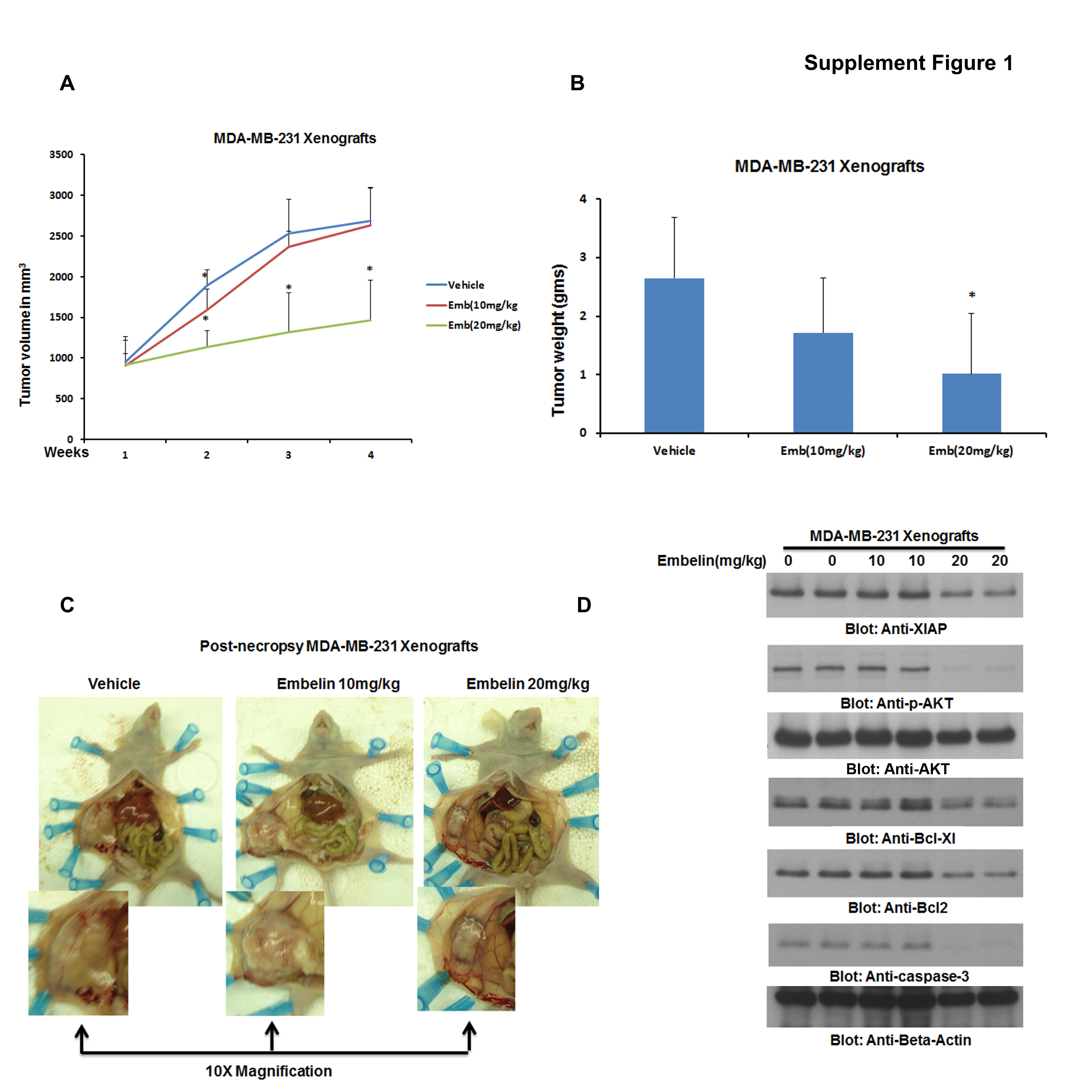

Supplement: Supplementary file 3 — Figure S1. Inhibition of PTC cell tumor-xenografts growth by embelin. Female nude mice at 6 weeks of age were injected subcutaneously with ten million MDA-MB-231 cells. After one week, the animals were randomly divided into three groups. The first groups were not treated and only vehicle (DMSO) was injected while the other two groups were treated 10 and 20 mg/kg embelin, injected intra-peritoneally, twice weekly for 4 weeks respectively. (A) The volume of each tumor was measured every week. The average (n = 4) tumor volume in each group of mice was calculated, * p < 0.05. (B) After 4 weeks treatment, mice were sacrificed and mean tumor weight (±SD) was calculated in each group. (C) Representative tumor images of each group of mice after necropsy. Inset showing 10X magnification. (D) Whole-cell homogenates from mice injected with TPC1cells were immuno-blotted with antibodies against XIAP, p-AKT, AKT, Bcl-Xl, Bcl-2 caspase 3 and beta-actin. (TIFF 9495 kb) [file 12885_2017_3627_MOESM3_ESM.tif]

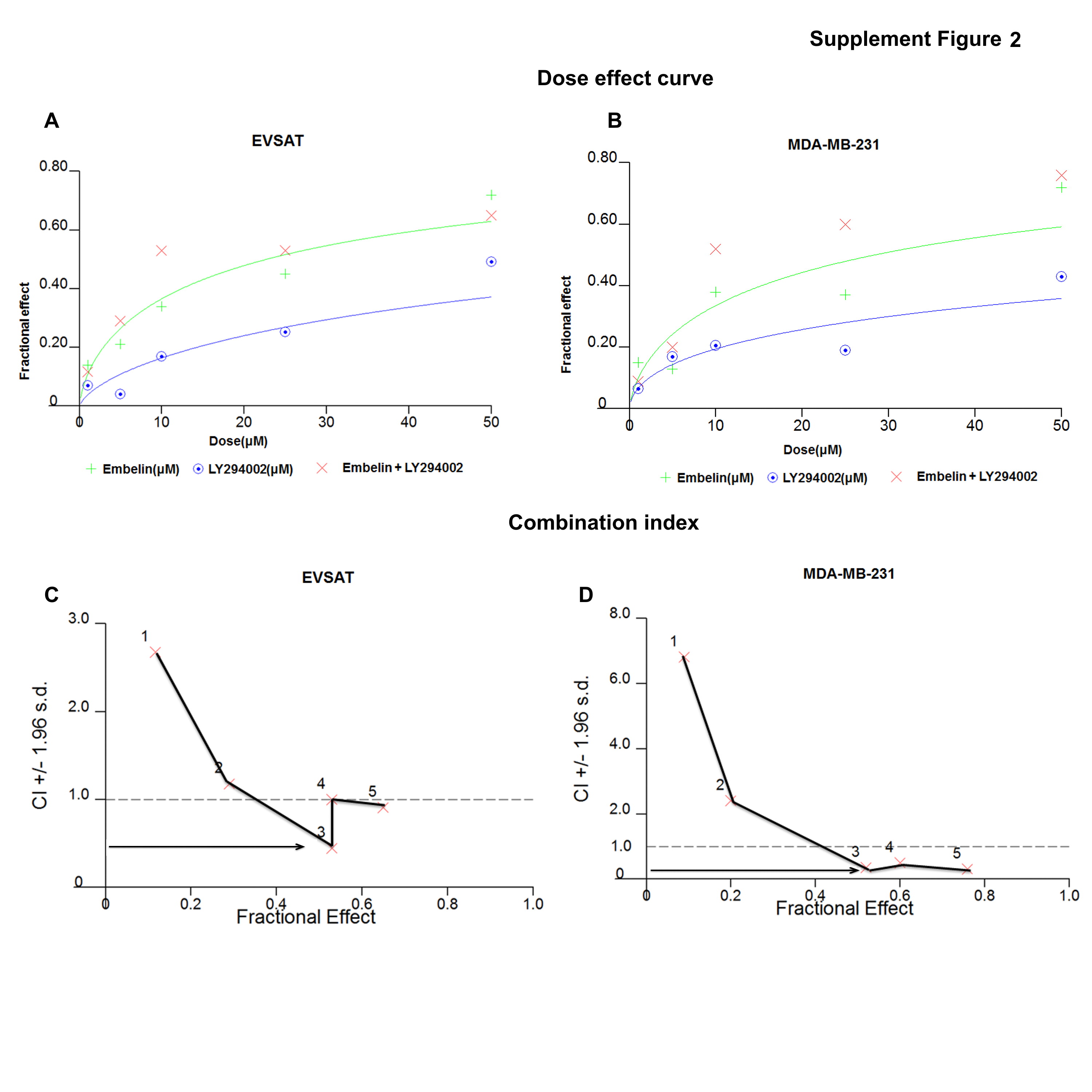

Supplement: Supplementary file 5 — Figure S2. Synergistic apoptotic response of embelin and LY294002 in BC cells. EVSAT and MDA-MB-231 cells were treated with various combinations of embelin and LY294002 for 24 h and dose effect (A and B) and Fractional effect (C and D) graphs were generated using Calcusyn software. Apoptotic response analysis was done as mean ± SD values normalized to control. Combination indices were calculated using Chou and Talalay methodology. (TIFF 1124 kb) [file 12885_2017_3627_MOESM5_ESM.tif]
