# Supplementary material for: XIAP over-expression is an independent poor prognostic marker in Middle Eastern breast cancer and can be targeted to induce efficient apoptosis
Source: BMC Cancer. 2017 Sep 11;17:640. doi: 10.1186/s12885-017-3627-4 (PMC5594504; doi:10.1186/s12885-017-3627-4)
Supplement: Supplementary file 4 — Table S3. Combination index calculation using Chou and Talalay method in BC cell lines. (DOCX 17 kb) [file 12885_2017_3627_MOESM4_ESM.docx]

**Supplementary Table 3: Combination index calculation using Chou and Talalay method in BC cell lines.**

**---------------------------------------------EVSAT-------------------------------------------------**

**Embelin LY294002 Fractional Combination index Dose Reduction Index**

**(μM) (μM) effect (Fa) (CI) (DRI)**

**% Apoptosis Embelin LY294002**

**(μM) (μM)**

**1 14.00**

**5.0 21.00**

**10.0 34.00**

**25 45.00**

**50 72.00**

**Median dose (Dm) 22.67μM**

**Exponent shape of curve (m) 0.67046 +/- 0.124949**

**Linear correlation coefficient (r) 0.95165**

**1.0 7.10 5.0 4.00 10 16.90**

**25 25.30**

**50 49.20**

**Median dose (Dm) 107.30µM**

**Exponent shape of curve (m) 0.68556 +/- 0.256496**

**Linear correlation coefficient (r) 0.83920**

**1. 1.0 10.0 11.70 2.67 1.11 0.56**

**2. 5.0 10.0 29.00 1.18 1.19 2.90**

**3. 10.0 10.0 53.00 0.447 12.78 12.78**

**4. 25.0 10.0 53.00 1.00 12.78 12.78**

**5. 50.0 10.0 65.00 0.914 26.47 26.47**

**----------------------------------------------MDA-MB-231------------------------------------------------**

**Embelin LY294002 Fractional Combination index Dose Reduction Index**

**(μM) (μM) effect (Fa) (CI) (DRI)**

**% Apoptosis** **Embelin LY294002**

**(μM) (µM)**

**1 15.00**

**5.0 13.00**

**10.0 38.00**

**25 37.00**

**50 72.00**

**Median dose (Dm) 28.33μM**

**Exponent shape of curve (m) 0.65391 +/- 0.222374**

**Linear correlation coefficient (r) 0.86164**

**1 6.50**

**5.0 17.00**

**10.0 20.60**

**25 19.00**

**50 43.00**

**Median dose (Dm) 152.8μM**

**Exponent shape of curve (m) 0.51991 +/- 0.119473**

**Linear correlation coefficient (r) 0.92911**

**1. 1.0 10.0 9.00 6.817 0.824 0.178**

**2. 5.0 10.0 20.00 2.411 0.680 1.062**

**3. 10.0 10.0 52.00 0.368 3.203 17.828**

**4. 25.0 10.0 60.00 0.505 2.107 33.337**

**5. 50.0 10.0 76.00 0.310 3.303 140.312**
